# Adaptable Hydrogels Mediate Cofactor‐Assisted Activation of Biomarker‐Responsive Drug Delivery via Positive Feedback for Enhanced Tissue Regeneration

**DOI:** 10.1002/advs.201800875

**Published:** 2018-10-22

**Authors:** Kunyu Zhang, Zhaofeng Jia, Boguang Yang, Qian Feng, Xiao Xu, Weihao Yuan, Xingfu Li, Xiaoyu Chen, Li Duan, Daping Wang, Liming Bian

**Affiliations:** ^1^ Department of Biomedical Engineering The Chinese University of Hong Kong Shatin, New Territories Hong Kong P. R. China; ^2^ Shenzhen Key Laboratory of Tissue Engineering Shenzhen Laboratory of Digital Orthopeadic Engineering Shenzhen Second People's Hospital (The First Hospital Affiliated to Shenzhen University, Health Science Center) Shenzhen 518035 P. R. China; ^3^ Guangdong Provincial Research Center for Artificial Intelligence and Digital Orthopedic Technology Shenzhen 518035 P. R. China; ^4^ Postgraduate institution Guangzhou Medical University Guangzhou 511436 P. R. China; ^5^ Shenzhen Research Institute The Chinese University of Hong Kong Shenzhen 518172 P. R. China

**Keywords:** adaptable hydrogels, drug delivery, positive feedback, tissue regeneration

## Abstract

The targeted and simultaneous delivery of diverse cargoes with vastly different properties by the same vehicle is highly appealing but challenging. Here, a bioactive nanocomposite hydrogel based on hyaluronic acid and self‐assembled pamidronate‐magnesium nanoparticles for the localized elution and on‐demand simultaneous release of bioactive ions and small molecule drugs is described. The obtained nanocomposite hydrogels exhibit excellent injectability and efficient stress relaxation, thereby allowing easy injection and consequent adaptation of hydrogels to bone defects with irregular shapes. Magnesium ions released from the hydrogels promote osteogenic differentiation of the encapsulated human mesenchymal stem cells (hMSCs) and activation of alkaline phosphatase (ALP). The activated ALP subsequently catalyzes the dephosphorylation (activation) of Dex phosphate, a pro‐drug of Dex, and expedites the release of Dex from hydrogels to further promote hMSC osteogenesis. This positive feedback circuit governing the activation and release of Dex significantly enhances bone regeneration at the hydrogel implantation sites. The findings suggest that these injectable nanocomposite hydrogels mediate optimized release of diverse therapeutic cargoes and effectively promote in situ bone regeneration via minimally invasive procedures.

## 1 Introduction

The controlled and long‐term delivery of therapeutic agents including protein‐based growth factors, small molecule drugs, genes, and bioactive ions is critical to the effective and efficient treatment of many pathological conditions.^1^ However, the simultaneous delivery of these diverse cargoes with vastly different properties by the same vehicle remains challenging.^2^ Hydrogels have a unique 3D cross‐linked polymeric network encompassing a wide range of chemical compositions and bulk physical properties and have been widely used in drug delivery applications.^3^ However, due to the intrinsic permeability and limited network interactions with cargo molecules in hydrogels, the sustained delivery is usually achieved on macromolecules like protein‐based growth factors, and the delivery of small‐molecule drugs and bioactive ions is still a challenge.

Synthetic glucocorticoids, such as dexamethasone (Dex), are widely used small‐molecule drugs in the clinical setting for purposes such as anti‐inflammation and immunosuppression.^4^ However, due to the rapid clearance, systematic therapies usually require long‐term and high‐dosage administration of glucocorticoids, and this may lead to many severe complications, such as osteoporosis or osteonecrosis.^5^ Therefore, smart delivery systems that enable localized delivery and triggered release of glucocorticoids will greatly boost the therapeutic efficacy while reducing the side effects due to systemic exposure. In addition, some glucocorticoids, such as Dex, can also induce differentiation of mesenchymal stem cells (MSCs) toward an osteogenic lineage and are thus widely used for stem cell‐based bone regeneration.^6^ To prevent multiple invasive treatments and simplify clinical procedures, a large bolus dose of Dex required for a course of treatment is usually applied. However, due to the lack of a reliable delivery approach in vivo, the initial burst release can lead to excessively high local concentration of Dex, which has been shown to severely inhibit osteogenesis and associated bone growth.^7^ The excess Dex may also suppress activities of macrophages and early immune response, which is required for tissue regeneration.^8^ These issues highlight once again the acute demand for a smart delivery system that can provide precisely controlled delivery of Dex in vivo.

Magnesium ions (Mg^2+^) play important roles in various biochemical processes and are one of the most important cofactors of many enzymes.^9^ Mg^2+^ is reported to be able to significantly enhance the adhesion and spreading of osteoblasts and promote the mineralization process.^10^ In our previous studies, we demonstrated that the nanocomposite hydrogels containing Mg^2+^ effectively enhanced the osteogenic differentiation of hMSCs and contributed to in situ bone regeneration.^11^ By further investigating the functions of Mg^2+^ in cellular metabolism, we noticed that it is also a critical cofactor for the enzymatic activity of alkaline phosphatase (ALP).^12^ ALP catalyzes the hydrolysis of monoesters of phosphoric acid to generate inorganic phosphate, which is essential for bone mineralization.^13^ Therefore, elevated levels of ALP can usually be observed at the site of bone defects/healing.^14^ Hence, ALP can be used as a regeneration‐specific trigger for mediating stimuli‐responsive delivery of drugs to promote bone healing.

**Scheme 1:**
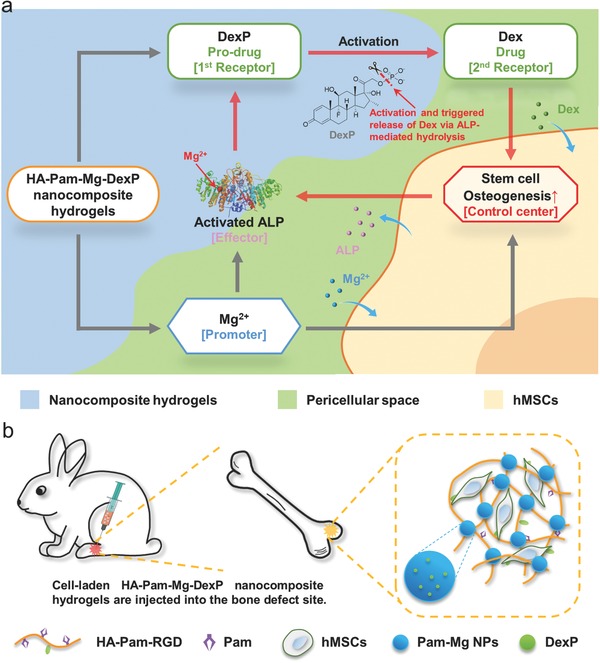
a) Schematic representation of “smart” hydrogels that mediate cofactor‐assisted activation of biomarker‐responsive drug delivery via a positive feedback loop (highlighted in red) for enhanced osteogenesis of encapsulated hMSCs. b) Injections of hMSC‐laden nanocomposite hydrogels promote in situ bone regeneration.

In this study, we developed a bioactive nanocomposite hydrogel to mediate the localized delivery and regeneration‐specific release of Dex. Hydrogels formed through the dynamic coordination between Mg^2+^ and pamidronate (Pam) exhibited excellent injectability and efficient stress relaxation, thereby allowing easy injection and consequent adaptation of hydrogels in bone defects with irregular shapes. Mg^2+^ released from the hydrogels can promote osteogenic differentiation of encapsulated hMSCs and activation of ALP. The self‐assembled nanostructure of Pam‐Mg^2+^ in the hydrogels stabilized Dex phosphate (DexP), a pro‐drug of Dex, in the hydrogel network via phosphate–Mg^2+^ interactions, thereby effectively reducing the nonspecific burst release of DexP from the hydrogels. At the bone healing sites, elevated ALP expression by osteogenically differentiated cells catalyzed the dephosphorylation (activation) of DexP and expedited the release of Dex due to lack of phosphate–Mg^2+^ interactions, and this can further promote osteogenesis of hMSCs (**Scheme** **1**a). This positive feedback circuit governing the activation and release of Dex from the hydrogels significantly enhanced bone regeneration at the hydrogel implantation sites (Scheme 1b). Our study provided a proof of concept for HA‐Pam‐Mg‐DexP hydrogels as an injectable and effective drug delivery platform to mediate optimized release of diverse therapeutic cargoes to promote the regeneration of anatomically deep and enclosed bone defects via minimally invasive procedures.

## 2 Results and Discussion

### 2.1 Fabrication of HA‐Pam‐Mg Nanocomposite Hydrogels

**Scheme 2:**
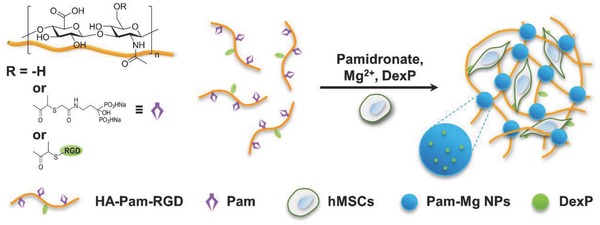
Schematic illustrations of the fabrication of self‐assembled HA‐Pam‐Mg nanocomposite hydrogels.

Hyaluronic acid (HA) is a key structural component in the native extracellular matrix that regulates multiple cellular behaviors of MSCs including proliferation and osteogenesis. Through a simple esterification reaction, we first synthesized methacrylated HA (MeHA) macromers (average degree of substitution, DS = 20% of HA repeating units) as the intermediate. The cell adhesion ligand, RGD peptide, was conjugated to HA backbone (DS = 3% of HA repeating units) to enhance cell–matrix interaction and facilitate cell spreading, especially in the 3D environment. The remaining methacryloyl groups were further used for conjugation of thiolglycolated pamidronate (thiol–Pam) via the thiol–ene click chemistry to yield pamidronate‐grafted HA (HA‐Pam) (Figure S1, Supporting Information). Simply mixing the HA‐Pam‐RGD macromer, Pam, and MgCl_2_ in phosphate buffered saline (PBS), leads to the formation of the self‐assembled Pam‐Mg nanoparticles (NPs), a process that was driven by the effective coordination between Pam and Mg^2+^. These Pam‐Mg NPs functioned as a cross‐linker to stabilize the HA‐Pam polymeric network, thereby resulting in the formation of HA‐Pam‐Mg nanocomposite hydrogels within 30 s. To encapsulate cells/drugs in the HA‐Pam‐Mg hydrogels, the cargoes (cells/drugs) were mixed with the hydrogel precursor prior to the addition of MgCl_2_ solution to induce gelation (**Scheme** **2**).

### 2.2 HA‐Pam‐Mg Nanocomposite Hydrogels Possess Tunable Mechanical Properties and Excellent Injectability

**Figure 1:**
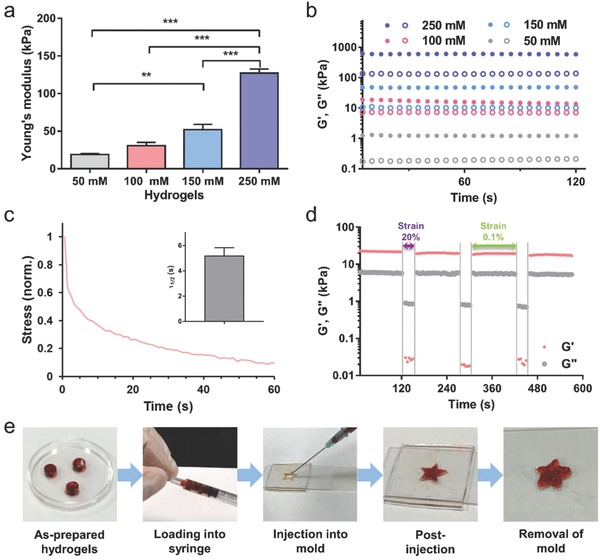
Mechanical properties of HA‐Pam‐Mg nanocomposite hydrogels. a) Average Young's modulus of HA‐Pam‐Mg*_x_* hydrogels (*n* = 3); *x* designates the concentration of Pam and MgCl_2_ (50, 100, 150, or 250 × 10^−3^ m); **p < 0.01, ***p < 0.001. b) Time sweep of dynamic rheology study of HA‐Pam‐Mg*_x_* hydrogels. c) Stress relaxation test for HA‐Pam‐Mg_100_ hydrogels under constant compressive strain. Inset: Quantification of timescale at which the stress was relaxed to half its original value, τ_1/2_ (*n* = 3). d) Rheological step‐strain oscillatory time‐sweep measurements displaying rapid recovery of the hydrogel structure under alternating high (20%) and low (0.1%) shear. e) Demonstration of injectability and moldability of the nanocomposite hydrogels.

The mechanical properties of HA‐Pam‐Mg nanocomposite hydrogels could be easily tuned by adjusting the concentrations of Pam and Mg^2+^ (*x*) while keeping the molar ratio between them at unity. As shown in **Figure** **1**a, with increasing *x*, the hydrogels became significantly stiffer (*E* = 18.83 ± 2.43 kPa for *x* = 50 × 10^−3^ m, and *E* = 127.17 ± 7.65 kPa for *x* = 250 × 10^−3^ m). Furthermore, increasing the concentration of Pam and Mg^2+^ also led to an increased storage and loss modulus of the hydrogels (Figure 1b), indicating that increasing the number of NP cross‐linkers could effectively stabilize the crosslinking networks. Subsequently, the hydrogels prepared by using 100 × 10^−3^ m of Pam and Mg^2+^ (HA‐Pam‐Mg_100_), with moderate mechanical properties, were used for further demonstrations and applications. As reported by Mooney and co‐workers, the fast relaxation rate (τ_1/2_ < 60 s) of hydrogels would be beneficial to the spreading and osteogenic differentiation of the encapsulated stem cells.^15^ Owing to the dynamic coordination between Pam and Mg^2+^, the relaxation time of the HA‐Pam‐Mg_100_ hydrogel was only 5.16 ± 0.65 s (Figure 1c), thereby suggesting that such hydrogels could provide a permissive mechano‐microenvironment for stem cells. This reversible nature of the dynamic coordination also contributed to the shear‐thinning property of the hydrogels. The step‐strain time‐sweep measurement revealed the rapid and full recovery of the hydrogel structure following repeated large deformations (Figure 1d). Moreover, because of the combination of the shear thinning behavior, excellent remoldability, and fast stress relaxation, HA‐Pam‐Mg hydrogels exhibited outstanding injectability and rapidly conformed to the geometry of the injection sites. As demonstrated in Figure 3e, the as‐prepared hydrogels could be loaded into a syringe and injected through a G21 needle into molds of different shapes and quickly adapted to the shape of the mold.

### 2.3 HA‐Pam‐Mg Nanocomposite Hydrogels Mediate Sustained Release of Mg^2+^ and ALP‐Triggered Release of Dex

**Figure 2:**
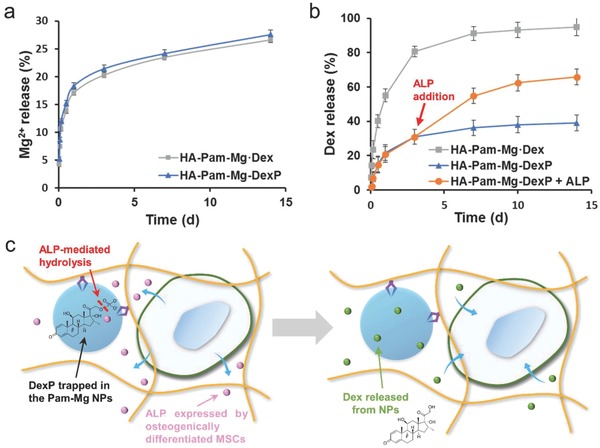
Sustained release of Mg^2+^ and Dex from the HA‐Pam‐Mg nanocomposite hydrogels. a) Cumulative release of Mg^2+^ from the HA‐Pam‐Mg hydrogels that were loaded with Dex via physical encapsulation (HA‐Pam‐Mg·Dex) or with DexP via the binding of DexP of Pam‐Mg NPs (HA‐Pam‐Mg‐DexP) in the PBS buffer (free of both Ca^2+^ and Mg^2+^). b) Cumulative release of Dex from the HA‐Pam‐Mg hydrogels in the PBS buffer. For ALP‐triggered Dex release, ALP (100 U mL^−1^) was added on day 3. c) Schematic illustrations of the biomarker‐responsive release of Dex from HA‐Pam‐Mg‐DexP hydrogels via positive feedback circuit.

To achieve the feedback‐regulated delivery of Dex, we loaded the HA‐Pam‐Mg hydrogels with DexP, during gelation. The putative coordination binding between the phosphate groups of DexP and Mg^2+^ helps integrate DexP molecules into the self‐assembled Pam‐Mg NPs. Scanning electron microscopy revealed densely packed Pam‐Mg NPs with the diameter of around 250 nm in the hydrogel networks (Figure S2, Supporting Information). Similar microstructures and stiffness (Figure S3, Supporting Information) of the DexP‐laden and blank hydrogels revealed that loading DexP did not significantly affect the microstructure of HA‐Pam‐Mg hydrogels, probably due to the substantial excess amount of Pam molecules compared to that of DexP (Pam: 100 × 10^−3^ m vs DexP: 20 × 10^−6^ m). We next investigated the release kinetics of Mg^2+^ from the nanocomposite hydrogels incubated in PBS (free of both Ca^2+^ and Mg^2+^) (**Figure** **2**a). The Mg^2+^ release profile of the DexP‐laden hydrogels (HA‐Pam‐Mg‐DexP) was similar to that of the control hydrogels containing phosphate‐free Dex, which was directly mixed into the hydrogel networks (HA‐Pam‐Mg·Dex). Both groups were able to sustain continuous release of Mg^2+^ during the two‐week release study despite an initial burst release. Notably, compared with the HA‐Pam‐Mg·Dex hydrogels, the HA‐Pam‐Mg‐DexP hydrogels exhibited a significantly smaller initial burst release and only limited subsequent release of Dex/DexP, indicating the effective trapping of DexP by the Pam‐Mg NPs. Furthermore, the addition of ALP induced a rapid and dose‐dependent release of Dex from the HA‐Pam‐Mg‐DexP hydrogels (Figure 2b, and Figure S4a, Supporting Information). The hydrolysis of the phosphate monoester bond and associated removal of the phosphate group in DexP catalyzed by ALP yielded the active Dex and expedited its release due to the lack of interaction between the dephosphorylated Dex and Pam‐Mg NPs. The DexP‐laden nanocomposite hydrogels were then incubated with various enzymes, such as trypsin and peroxidase, under identical conditions. As shown in Figure S4b (Supporting Information), ALP induced significantly more Dex release than the other enzymes, indicating that this drug delivery system has high specificity toward ALP. It is known that the expression of ALP is significantly elevated at the locations of bone growth/healing,^14^ and we expected that this expressed ALP would trigger the on‐demand release of Dex at the specific sites where Dex delivery was desired. The released Dex could further promote osteogenesis of stem/progenitor cells and subsequently resulted in more ALP expression (Figure 2c). Therefore, these cascading events constituted the positive feedback loop that enabled targeted local release of Dex from HA‐Pam‐Mg‐DexP hydrogels to enhance osteogenesis and bone regeneration (Scheme 1a).

### 2.4 HA‐Pam‐Mg Hydrogels Induce Elevated ALP Activity of hMSCs via Released Mg^2+^

**Figure 3:**
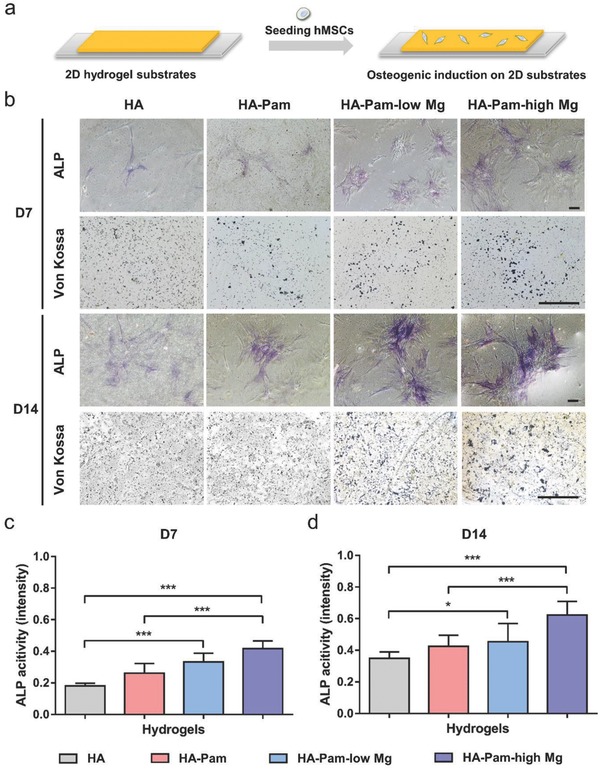
Osteogenesis and ALP activity of hMSCs seeded on 2D hydrogel substrates loaded with varying dosage of Mg^2+^ (low: 10 × 10^−3^ m, high: 100 × 10^−3^ m). a) Schematic illustrations of seeding hMSCs on 2D hydrogel substrates. b) Alkaline phosphatase and von Kossa staining of hMSCs cultured on 2D hydrogels substrates after 7 or 14 d of osteogenic induction; scale bar = 100 µm. c,d) Average ALP activity of hMSCs on hydrogels after 7 or 14 d, respectively (*n* = 30); *p < 0.05, ***p < 0.001.

Mg^2+^ has been shown to enhance the osteogenic differentiation of stem cells and is an important regulator in the bone healing process.^16^ Herein, we investigated the osteogenesis and ALP biosynthesis of hMSCs cultured on the 2D substrates of HA‐Pam‐Mg hydrogels fabricated with varying dosage of Mg^2+^ (low: 10 × 10^−3^ m, high: 100 × 10^−3^ m) (**Figure** **3**a). RGD peptide was conjugated to all groups of the hydrogel substrates for further enhancement of cell attachment, especially for the Mg^2+^‐free groups. After 7 or 14 d of osteogenic induction, although the phosphate‐containing hydrogels, for example, HA‐Pam, were reported previously to be able to promote osteogenesis and calcification,^17^ the von Kossa staining still showed more calcium deposition in the Mg^2+^‐containing groups (HA‐Pam‐low Mg, HA‐Pam‐high Mg) than the control groups without Mg^2+^ (HA, HA‐Pam), thereby proving that Mg^2+^ released from the hydrogels can further promote osteogenesis (Figure 3b). Moreover, staining against ALP, indeed showed significantly higher ALP activity in the hMSCs cultured on the Mg^2+^‐containing hydrogels (HA‐Pam‐low Mg, HA‐Pam‐high Mg) than that of the control groups (HA, HA‐Pam) (Figure 3b). Furthermore, cells on the hydrogels containing higher concentration of Mg^2+^ exhibited even higher ALP activity compared to those on the hydrogels containing lower Mg^2+^ concentrations, indicating that Mg^2+^‐induced enhancement of ALP activity was dose‐dependent (Figure 3c,d). Elevated ALP activity would further induce the release of Dex through a positive feedback cycle. Furthermore, Mg^2+^ is also a critical cofactor that is vital to the activity of ALP. Therefore, through these multiple pathways, the Mg^2+^ released from the hydrogels could simultaneously enhance ALP expression and activity and served as a promoter in this positive feedback circuit (Scheme 1a).

### 2.5 HA‐Pam‐Mg‐DexP Hydrogels Enhance Osteogenesis of hMSCs

To further assess the effect of the Dex feedback release on cell behaviors in 3D, we encapsulated hMSCs in HA‐Pam‐Mg·Dex (physical encapsulation of Dex) or HA‐Pam‐Mg‐DexP (DexP incorporated via phosphate–Mg^2+^ coordination) hydrogels and cultured them in Dex‐free osteogenic medium. The cells encapsulated in Dex‐free hydrogels but cultured in Dex‐supplemented medium (HA‐Pam‐Mg&Dex) were used as a positive control group. The amount of Dex/DexP loaded in HA‐Pam‐Mg·Dex and HA‐Pam‐Mg‐DexP groups was determined by the cumulative amount of Dex supplemented in the HA‐Pam‐Mg&Dex group. After 7 or 14 d of culture, the majority (>90%) of hMSCs in all groups remained viable (Figures S6 and S7a,b, Supporting Information), indicating that all of the hydrogels were cytocompatible.

**Figure 4:**
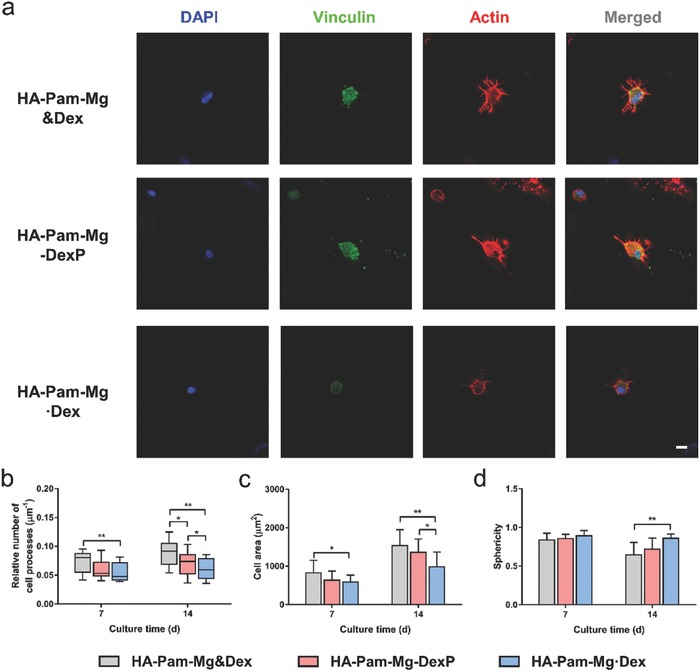
a) Fluorescent staining of hMSCs encapsulated in the 3D hydrogels after 14 d of osteogenic induction; scale bar = 10 µm. Quantitative analyses of b) relative number of cell processes per µm of cell perimeter, c) cell area, and d) cell shape factors of hMSCs encapsulated in the 3D hydrogels after 7 or 14 d of osteogenic induction; *p < 0.05, **p < 0.01, ***p < 0.001.

In the 3D environment, a cell explores its surroundings by extending cell membrane processes, which are driven by the assembly of cytoskeletal structure. Fluorescent staining demonstrated that hMSCs encapsulated in all of the hydrogels developed protrusions from the edges of cell membrane after 7 or 14 d of culture, likely owing to the highly dynamic structure of the nanocomposite hydrogels (**Figure** **4**a, and Figure S8, Supporting Information). Specifically, cells in the HA‐Pam‐Mg&Dex and HA‐Pam‐Mg‐DexP groups developed significantly more cell‐membrane processes, compared to those in the HA‐Pam‐Mg·Dex group (Figure 4b). We also observed that cells in the HA‐Pam‐Mg·Dex group mainly maintained a spherical morphology, whereas cells in the former two groups exhibited more isotropic spreading, as evidenced by the larger cell area and smaller sphericity (Figure 4c,d). It has been reported that Dex can stimulate expression of fibronectin and integrin at cell adhesion sites, which are essential to cell spreading and generation of cellular tension.^18^ Thus, sustained supply of Dex in the HA‐Pam‐Mg&Dex and HA‐Pam‐Mg‐DexP group may promote the cellular adhesion and interaction with the hydrogel network, thereby facilitating the spreading of encapsulated cells.

**Figure 5:**
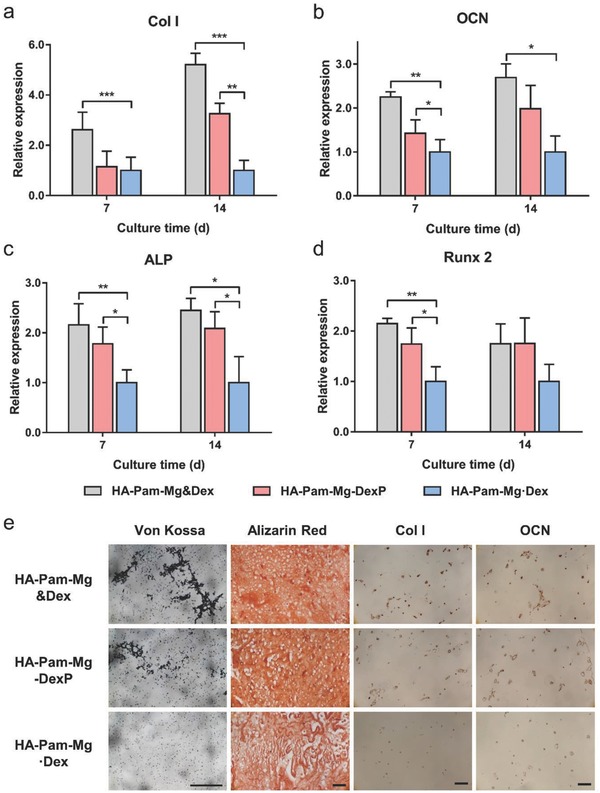
HA‐Pam‐Mg nanocomposite hydrogels promote osteogenesis of encapsulated hMSCs. Relative gene expression of selected osteogenic markers a) type I collagen (“Col I”), b) osteocalcin (“OCN”), c) alkaline phosphatase (“ALP”), and d) runt‐related transcription factor 2 (“Runx 2”) after 7 or 14 d of osteogenic culture, respectively (*n* = 3); *p < 0.05, **p < 0.01, ***p < 0.001. e) Von Kossa staining, alizarin red staining, and immunohistochemical staining of type I collagen (“Col I”) and osteocalcin (“OCN”) of the hMSC‐laden hydrogels after 14 d of osteogenic culture; scale bar = 100 µm.

Cell spreading is closely correlated with the development of actomyosin contractility, mechanosensing, and osteogenic differentiation of stem cells. The different morphologies displayed by the encapsulated hMSCs in different groups indicate different level of activation of mechanotransduction signaling and associated cellular behaviors, especially osteogenic differentiation.^19^ Our real‐time polymerase chain reaction (RT‐PCR) analysis revealed the highest expression of osteogenic marker genes, including type I collagen, osteocalcin, and Runx 2, in the positive control group (HA‐Pam‐Mg&Dex) with continuous media supplementation of Dex (**Figure** **5**a–d). Compared with the Dex‐laden group (HA‐Pam‐Mg·Dex), the DexP‐laden group (HA‐Pam‐Mg‐DexP) showed significantly upregulated mRNA expression of these markers. For example, the expression level of ALP in the HA‐Pam‐Mg‐DexP group was 78.0% higher than that of HA‐Pam‐Mg·Dex group at Day 7 (Figure 5c). The HA‐Pam‐Mg‐DexP hydrogels had a similar content of calcification compared to the HA‐Pam‐Mg&Dex group, and both groups had significantly higher calcium content than the HA‐Pam‐Mg·Dex group (Figure S7c, Supporting Information). A similar trend was also revealed by the von Kossa and alizarin red staining of hydrogel histological sections (Figure 5e). Furthermore, as evidenced by the immunohistochemical staining, the HA‐Pam‐Mg&Dex and HA‐Pam‐Mg‐DexP groups also showed much more intense staining against type I collagen and osteocalcin than the HA‐Pam‐Mg·Dex group (Figure 5e). Therefore, although the release of Mg^2+^ may lead to the softening of the hydrogels (Figure S5, Supporting Information), the deposition of calcium and accumulation of collagen fibers may help maintain the bulk stiffness of the hydrogels during culture (Figure S9, Supporting Information). It is known that Dex can promote the lineage commitment of hMSCs to osteoblasts by enhancing RhoA activities.^20^ Our results indicated that the HA‐Pam‐Mg‐DexP hydrogels mediated sustained release of Dex via the ALP‐triggered positive feedback and further promoted the osteogenic differentiation of encapsulated hMSCs. Therefore, our hydrogels should prove to be an ideal carrier for the delivery of stem cells together with the differentiation‐inducing drugs to tissue defects in vivo, where the continuous addition of drugs is usually impractical.

### 2.6 HA‐Pam‐Mg‐DexP Hydrogels Promote In Situ Bone Regeneration

**Figure 6:**
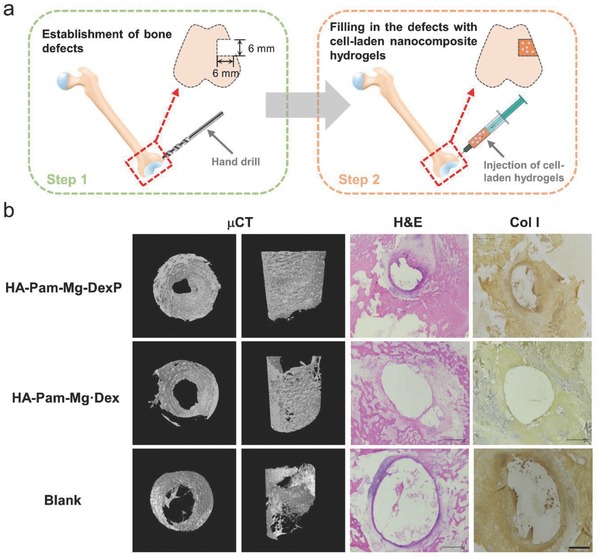
HA‐Pam‐Mg nanocomposite hydrogels encapsulating MSCs promote healing of rabbit femur defects. a) Schematic illustration of the bone defect establishment at the end of lateral femur epicondyle by a hand drill and the injection of HA‐Pam‐Mg nanocomposite hydrogels into the defects. b) Micro‐CT reconstruction images, H&E staining, and type I collagen (“Col I”) immunohistochemical staining of the histological sections of rabbit femurs eight weeks after surgery.

Having established the ability of the HA‐Pam‐Mg‐DexP hydrogels to direct the osteogenic differentiation of stem cells, we next examined their performance to drive bone regeneration in vivo. Rabbit MSCs were encapsulated in the hydrogels and then immediately injected into rabbit femur defects (**Figure** **6**a). A sham treatment group receiving saline injection (Blank) was included as the control group. Eight weeks after implantation, microcomputed tomography (μCT) analyses revealed that dense calcified tissue was formed within the defects treated with HA‐Pam‐Mg‐DexP hydrogels, while the growth of neo‐bone in the control groups (Blank, HA‐Pam‐Mg‐Dex) was retarded (Figure 6b). Quantitative analysis showed significantly more bone volume (normalized to the total volume) in the DexP‐laden group than in the control groups (Figure S10, Supporting Information). The newly formed bony tissue was also analyzed histologically through hematoxylin and eosin (H&E) staining and type I collagen immunohistochemical staining (Figure 6b). Bone defects in the blank control group were still clearly visible, whereas bone defects implanted with the nanocomposite hydrogels were filled with significantly more bone tissue. In particular, the defects implanted with the HA‐Pam‐Mg‐DexP hydrogels were mostly healed. The lack of the on‐demand smart release of induction factors was one of the critical reasons that restricted the in vivo performance of many biomaterials. These promising results illustrated that our nanocomposite hydrogel could achieve sustained release of Mg^2+^ and Dex in vivo in response to cellular markers in a feedback‐controlled fashion and effectively stimulate in situ bone regeneration. Additionally, bone regeneration is also highly dependent on angiogenesis, that is, formation of new blood vessels around the injury site.^21^ Although BPs have been shown to stimulate osteoblast proliferation and differentiation and inhibit apoptosis, reports in recent year have raised concerns about the suppressed angiogenesis, a potential complication following long‐term treatment with BPs.^22^ Therefore, the loading amount and release kinetics of BPs in the hydrogels should be carefully tailored. Meanwhile, the cotreatment with calcitriol may partially reverse this bisphosphonate‐induced inhibition;^23^ therefore, a codelivery system can be designed in the future works to limit the potential side effects of bisphosphonate delivery.

## 3 Conclusion

Taken together, we have presented a self‐assembled HA‐Pam‐Mg nanocomposite hydrogel with tunable mechanical properties, excellent injectability, rapid stress relaxation, and unique bioactivities. Owing to the interaction between Mg^2+^ and DexP, this small molecular prodrug can be stably loaded into the hydrogels with low basal release and become activated and initiate expedited release in response to a regeneration marker, ALP. The Mg^2+^ released from the hydrogels can promote osteogenic differentiation of the encapsulated hMSCs and activation of ALP. Meanwhile, the elevated ALP expression and activity promoted the activation and release of Dex from the hydrogels, thereby further enhancing the osteogenesis of hMSCs. Therefore, this positive feedback circuit of drug release regulation from hydrogels can significantly enhance bone regeneration at the intended sites. Our findings demonstrated the promising potential of our HA‐Pam‐Mg nanocomposite hydrogels as carriers of therapeutic cells and drugs for bone repair by minimally invasive procedures.

## 4 Experimental Section

*Materials*: Sodium hyaluronate (*M*_W_ = 80 kDa) was purchased from Bloomage Freda Biopharm (China). Pamidronate disodium salt (Pam) was purchased from Dalian Meilun Biology Technology (China). Magnesium chloride hexahydrate and sodium hydroxide were obtained from Aladdin Reagent (China). Methacrylic anhydride, propidium iodide (PI), paraformaldehyde, Triton X‐100, 4′,6‐diamidino‐2‐phenylindole (DAPI), sliver nitrate, and sodium thiosulfate were ordered from Sigma‐Aldrich (USA). Deuterium oxide (D_2_O), N‐(3‐dimethylaminopropyl)‐N′‐ethylcarbodiimide hydrochloride (EDC), and N‐hydroxysuccinimide (NHS) were obtained from the J&K Scientific (China). All chemicals were used as received without further purification. PBS, α‐minimum essential medium (α‐MEM), penicillin/streptomycin (pen/strep), l‐glutamine, calcein AM, and fetal bovine serum (FBS) were obtained from Gibco (USA). Alkaline phosphatase was purchased from Baomanbio (China). Magnesium colorimetric assay kit and calcium colorimetric assay kit were purchased from Bio Vision (USA). BCA protein assay kit and revertAid First strand cDNA synthesis kit were obtained from Thermo (USA). Peroxidase substrate kit DAB and vectastain ABC kit were purchased from Vector Lab (USA). Human mesenchymal stem cells (hMSCs) were obtained from Lonza. The water used in all the experiments was purified by Millipore system.

*Synthesis of MeHA and HA‐Pam‐RGD*: Methacrylated HA (MeHA) was synthesized as previously reported.^24^ Pamidronate disodium salt, thioglycolic acid, EDC, and NHS were dissolved in NaOH solution (pH = 8.0) together and stirred for reaction. The crude product was precipitated from water upon the addition of absolute ethanol. The precipitate (thiol–Pam) was collected by centrifugation and washed with ethanol for several times. The cell adhesion ligand, RGD peptide (GCGYGRGDSPG), was conjugated to HA backbone (DS = 3% of HA repeating units) via the thiol–ene click chemistry in alkaline phosphate buffer (0.2 m Na_2_HPO_4_‐NaH_2_PO_4_, pH = 8.0) with tcep (5 × 10^−3^ m). The remaining methacryloyl groups were further used for conjugation of thiolglycolated pamidronate (thiol–Pam) in TEOA buffer solution (pH = 8.5, with 5 × 10^−3^ m tcep) with continuously stirring for 48 h. The mixture was dialyzed against NaCl solution and deionized (DI) water for 3 d, respectively, then frozen at −80 °C, lyophilized and stored at −20 °C in powder form.

*Fabrication of HA‐Pam‐Mg Injectable Nanocomposite Hydrogels*: HA‐Pam‐RGD (2% w/v) and Pam (100 × 10^−3^ m) were dissolved in PBS, and the hydrogels were then formed upon the addition of MgCl_2_ stock solution during vertex. For the hydrogels containing Dex or DexP, the drugs were mixed with the precursor prior to the addition of MgCl_2_.

*Compression Test*: Compression test of the hydrogels was performed on the rheometer (Malvern KINEXUS Lab+). The cylindrical hydrogel samples (*d* = 4 mm, *h* = 2 mm) were prepared in advance and equilibrated in PBS buffer. The compressive strain rate was set at 0.05 mm min^−1^, and samples were compressed to failure. All tests were done on triplicate samples (*n* = 3).

*Rheological Measurement*: Rheological measurements of the materials were performed using a rotational rheometer (Malvern KINEXUS Lab+) with 4 mm diameter of plate geometry. For oscillatory time sweep experiments, the storage (*G*′) and loss (*G*″) moduli were measured under constant strain (0.1%) and frequency (1 Hz). For the characterization of the shear thinning properties, the hydrogel samples were subjected to sequential shear strains of 0.1 and 20% for several cycles, and the recovery of storage and loss moduli were monitored by time sweeps with fixed frequency (1 Hz).

*Quantification of the Release of Magnesium Ions, Dex or DexP from Hydrogels*: To investigate the release rate of Mg^2+^ and dexamethasone from the nanocomposite hydrogels, the hydrogel samples were incubated in 350 µL of Ca^2+^/Mg^2+^‐free PBS buffer at 37 °C. For enzyme‐responsive Dex release, ALP (100 U mL^−1^) was added to the samples on day 3. All the PBS was collected and replenished at each preset time points. All samples were in triplicate (*n* = 3). The amount of Mg^2+^ released was analyzed by magnesium colorimetric assay kit according to manufacturer's protocol, and the absorbance at 242 nm was measured for the determination of Dex or DexP.

*Osteogenic Differentiation of hMSCs on 2D Substrates*: The 2D hydrogel substrates containing Pam and Mg^2+^ were prepared as previously reported, and the RGD peptide was conjugated to further improve the cell attachment.^11^ The mechanical stiffness of the substrates was controlled to be similar by adjusting MeHA concentration. hMSCs were expanded to passage 4 and then seeded on the hydrogel substrates. Cells were cultured in the osteogenic medium (α‐MEM, 16.67% FBS, 1% glutamine, 1% pen/strep, 10 × 10^−3^ m β‐glycerophosphate disodium, 50 mg mL^−1^ ascorbate, 0.1 × 10^−6^ m dexamethasone), and medium was changed three times per week. After osteogenic induction for 7 or 14 d, the seeded cells were fixed with 4% paraformaldehyde, rinsed in PBS for several times, and permeabilized with 0.25% Triton X‐100 in PBS for 30 min at room temperature. Alkaline phosphatase activity was stained by Fast Blue staining, and tissue calcification was stained by von Kossa staining.

*Encapsulation and Osteogenic Differentiation of hMSCs in Nanocomposite Hydrogels In Vitro*: For 3D cell culture, hMSCs were encapsulated in the hydrogels at 1 × 10^7^ mL^−1^ and cultured in the osteogenic medium. Media change was performed three times per week. Cell viability was assessed by using a Live/Dead assay, in which live cells were stained green with calcein‐AM while dead cells were stained red with propidium iodide.

*Fluorescent Staining and Analysis*: Cells were fixed with 4% paraformaldehyde solution for 20 min at room temperature, permeabilized in 0.25% Triton‐X 100 in PBS for 1 h and blocked with 2.5% BSA in PBS for another 1 h. For morphology studies, the cytoskeleton was stained with phalloidin‐TRITC, and cell nuclei were stained with DAPI. For immunofluorescent staining of vinculin, cells were incubated with the primary antibody at 4 °C overnight, followed by the secondary antibody conjugated with FITC. Fluorescent images were acquired with a Nikon C2+ confocal microscope and analyzed using Image J (NIH). For quantification of the number of cell processes, cell processes were defined as thin (<1 µm wide) phalloidin‐stained protrusions from the cell margin. More than ten microscope fields were captured for each group.

*Gene Expression Analysis*: For gene expression analysis, samples were harvested and homogenized in Trizol reagent, and whole RNA was extracted according to the manufacturer's instructions. The RNA concentration was then determined by using a Nanodrop One spectrophotometer (Nanodrop Technologies). 100 ng of RNA from each sample was reverse transcribed into cDNA using RevertAid Fist Strand cDNA Synthesis Kit (Thermo). Real‐time PCR was performed on an Applied Biosystems StepOnePlus Real‐Time PCR system using Taqman primers and probes specific for GAPDH (housekeeping gene) and other osteogenic marker genes including type I collagen, osteocalcin, alkaline phosphatase, and runt‐related transcription factor 2. The relative gene expression was calculated using the ΔΔC_T_ method, where fold difference was calculated using the expression $2^{-\Delta \Delta C_{T}}$.

*Rabbit Bone Defect Regeneration*: Skeletally mature female New Zealand white rabbits (2.0–2.5 kg, 5–6 months old) were anesthetized and draped for aseptic surgery. The rabbit was placed in a dorsal‐recumbent position and a vertical longitudinal incision was made at the midpoint of the lateral femoral condyle. A cylindrical bone defect was created parallel to the articular surface (6 mm in both diameter and depth) using a hand drill and rinsed with saline repeatedly. The cell‐laden HA‐Pam‐Mg hydrogels containing Dex or DexP were then injected into the defects, and the soft tissues were closed layer by layer. Blank group received no implants and was used as the control group. Rabbits were sacrificed after eight weeks, and samples were harvested and fixed by 4% paraformaldehyde for the following μCT and histological analyses. All the animal experiments were conducted in accordance with the regional Ethics Committee guidelines, and all animal procedures were approved by the Institutional Animal Care and Use Committee of Shenzhen Second People's Hospital, Shenzhen University.

*Biochemical Analysis*: One‐half of each hydrogel sample (two samples for each group) was crushed and incubated in 100 µL of 1 m HCl overnight, followed by neutralization via the addition of 5 m NaOH. The BCA and calcium content of each sample were measured according to manufacturer's instruction, and the calcium content was then normalized by BCA content.

*Histological Analysis*: Samples were embedded in paraffin in accordance with the standard histological procedures. The histological sections (8 µm for hydrogel samples and 5 µm for decalcified bone samples) were stained for targets of interest. H&E stain was prepared following the manufacturers' instructions. For type I collagen and osteocalcin immunochemical staining, the sections were stained by using the Vectastain ABC kit with the DAB Substrate kit for peroxidase. Nonimmune controls were processed following the same procedure without primary antibody incubation. Sections were also stained for calcification by using Von Kossa staining and Alizarin Red S staining.

*Statistical Analysis*: All data are presented as mean ± standard deviation. Statistical analysis was performed by using one‐way ANOVA and Tukey's post hoc testing. Tests were conducted with a 95% confidence interval (α = 0.05).

## Conflict of Interest

The authors declare no conflict of interest.

## Supporting information

Supplementary
