# Supplementary material for: Adaptable Hydrogels Mediate Cofactor‐Assisted Activation of Biomarker‐Responsive Drug Delivery via Positive Feedback for Enhanced Tissue Regeneration
Source: Adv Sci (Weinh). 2018 Oct 22;5(12):1800875. doi: 10.1002/advs.201800875 (PMC6299823; doi:10.1002/advs.201800875)
Supplement: Supplementary file 1 — Supplementary [file ADVS-5-1800875-s001.pdf]

Copyright WILEY-VCH Verlag GmbH & Co. KGaA, 69469 Weinheim, Germany, 2018.

ADVANCED SCIENCE Open Access

Supporting Information

for *Adv. Sci.,* DOI: 10.1002/advs.201800875

Adaptable Hydrogels Mediate Cofactor-Assisted Activation of Biomarker-Responsive Drug Delivery via Positive Feedback for Enhanced Tissue Regeneration

Kunyu Zhang, Zhaofeng Jia, Boguang Yang, Qian Feng, Xiao Xu, Weihao Yuan, Xingfu Li, Xiaoyu Chen, Li Duan, Daping Wang, and Liming Bian**

Copyright WILEY-VCH Verlag GmbH & Co. KGaA, 69469 Weinheim, Germany, 2016.

Supporting Information

Adaptable hydrogels mediate cofactor-assisted activation of biomarker-responsive drug delivery via positive feedback for enhanced tissue regeneration

Kunyu Zhang[†]*, Zhaofeng Jia*[†]*, Boguang Yang, Qian Feng, Xiao Xu, Weihao Yuan, Xingfu Li, Xiaoyu Chen, Li Duan, Daping Wang* and Liming Bian**

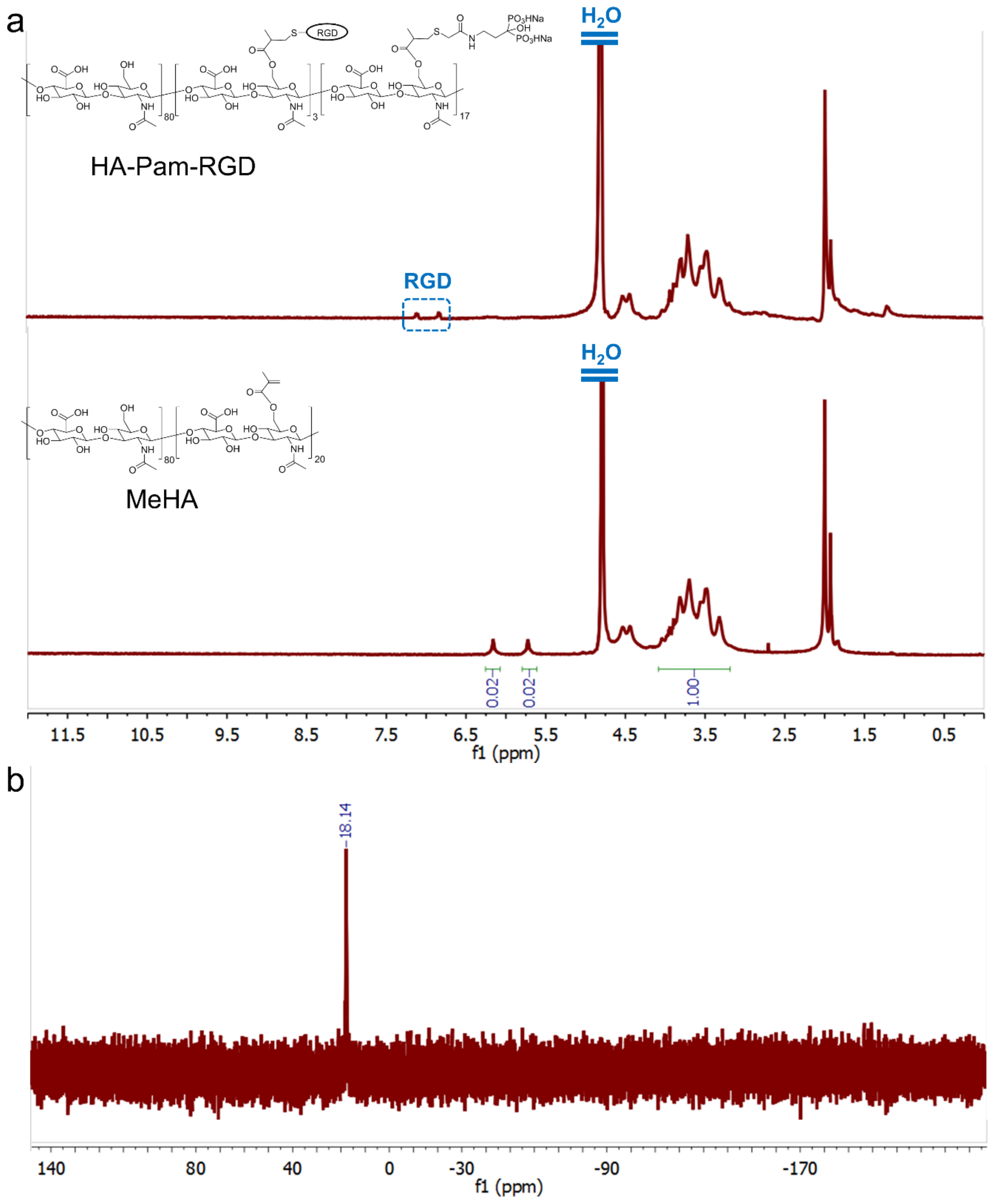

Figure S1. a) ^{1}H NMR spectra of MeHA and HA-Pam-RGD, indicating the complete consumption of the methacryloyl groups in MeHA. b) ^{31}P NMR spectrum of HA-Pam-RGD, confirming the successful conjugation of Pam to HA.

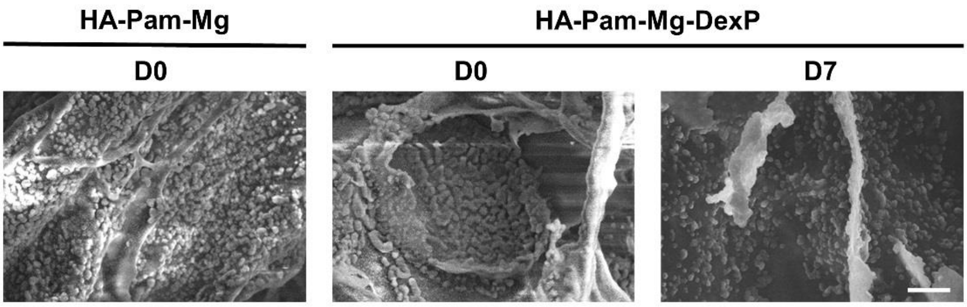

Figure S2. SEM images of the nanocomposite hydrogels; scale bar = 2.5 μm.

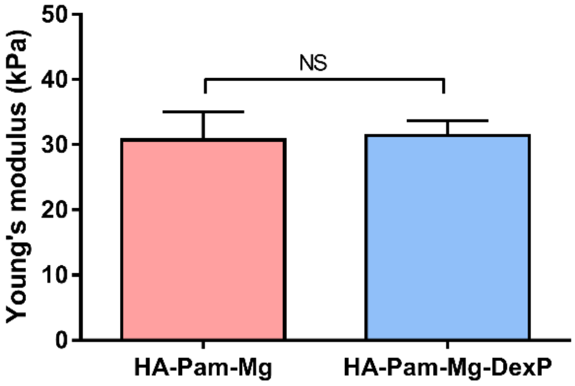

Figure S3. Average Young's modulus of unloaded and DexP-laden HA-Pam-Mg nanocomposite hydrogels. "NS" indicates no statistical significance ($P > 0.05$).

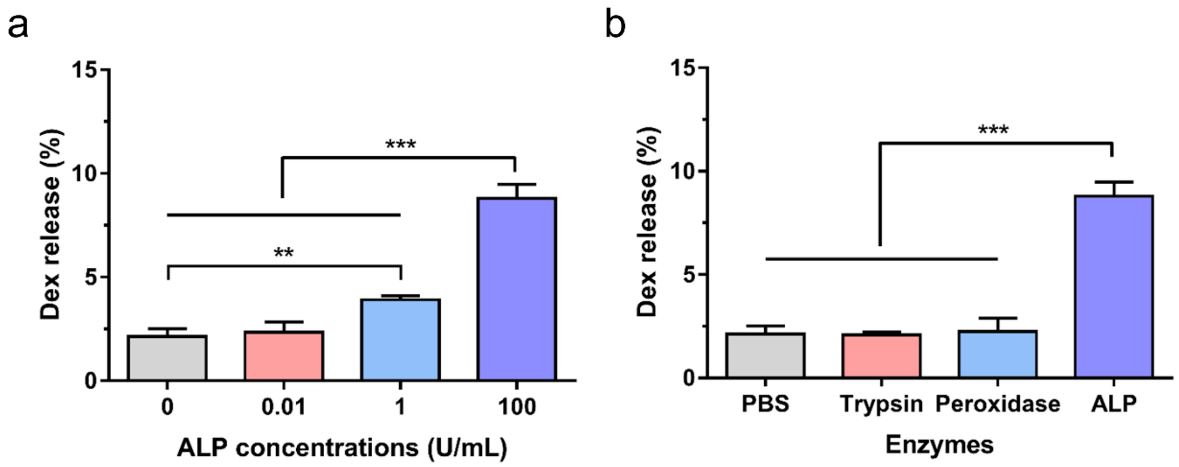

Figure S4. Dex release from the nanocomposite hydrogels after 24-hour incubation with a) various concentrations of ALP or b) various enzymes (100 U/mL). The DexP-laden hydrogels were pre-equilibrated in PBS for 3 days. **$p < 0.01$, ***$p < 0.01$.

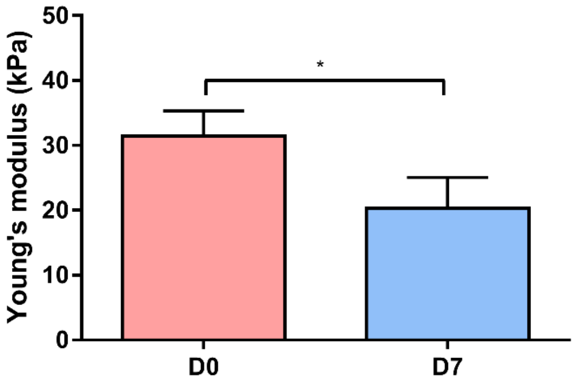

Figure S5. Average Young's modulus of the DexP-laden HA-Pam-Mg nanocomposite hydrogels before/after incubation in PBS for 7 days; *$p < 0.05$.

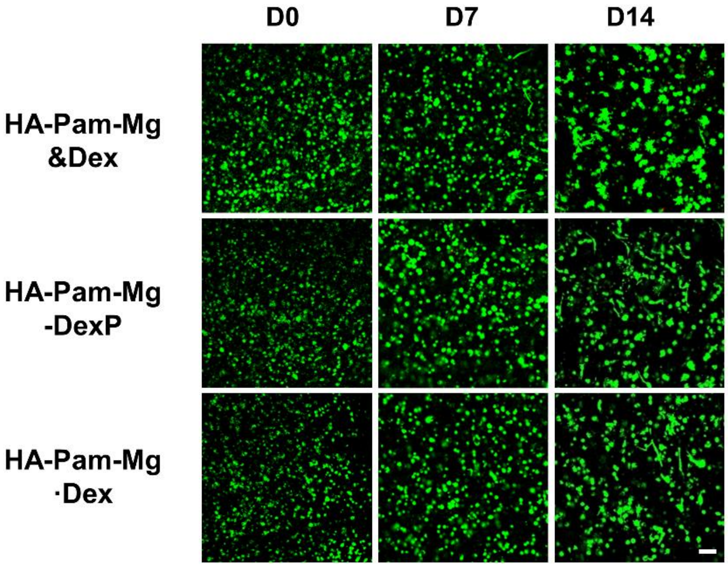

Figure S6. Live/dead staining (green: live, red: dead) of hMSCs encapsulated in the nanocomposite hydrogels; scale bar = 200 μm.

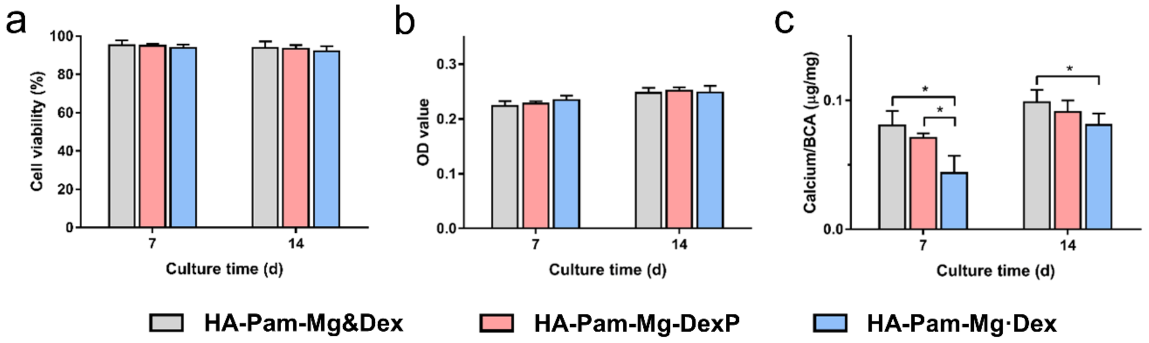

Figure S7. a) Cell viability of hMSCs encapsulated in the nanocomposite hydrogels as determined by live/dead staining. b) Alamar Blue assay showed that the encapsulated hMSCs exhibited similar levels of metabolic activity in all groups (n = 3). c) Calcium content of the cell-laden nanocomposite hydrogels (normalized to total protein content) (n = 3); *$p < 0.05$.

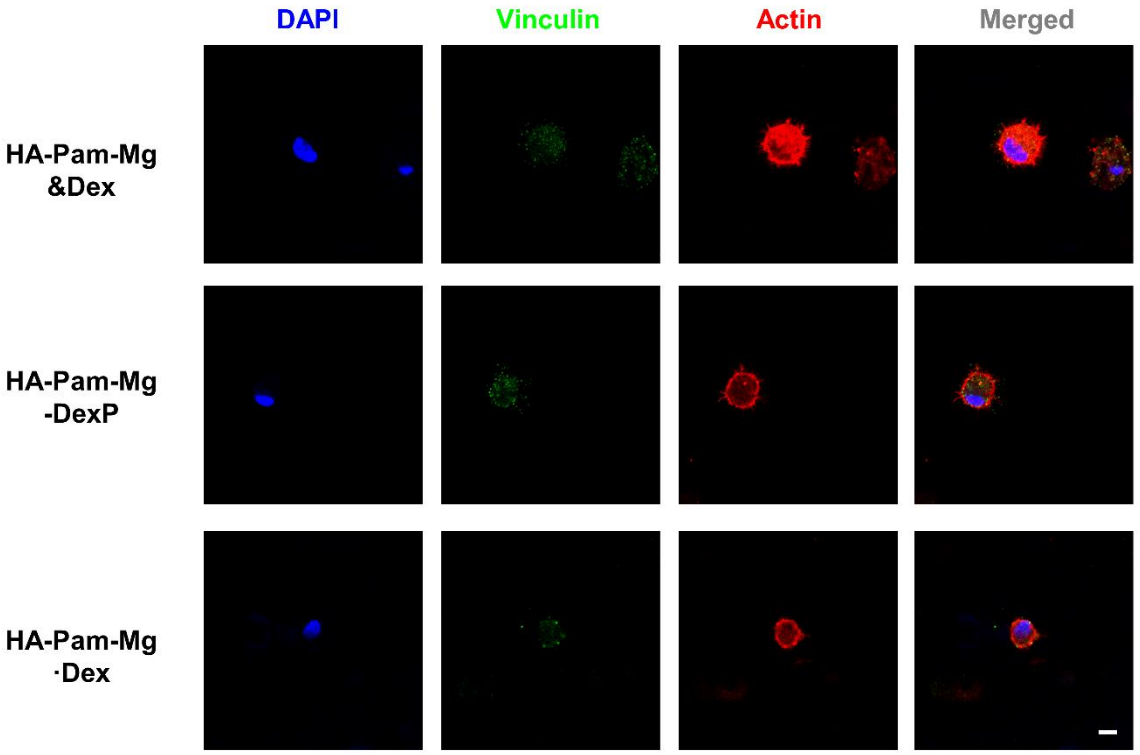

Figure S8. Fluorescent staining of hMSCs encapsulated in the 3D hydrogels after 7 days of osteogenic induction; scale bar = 10 μm.

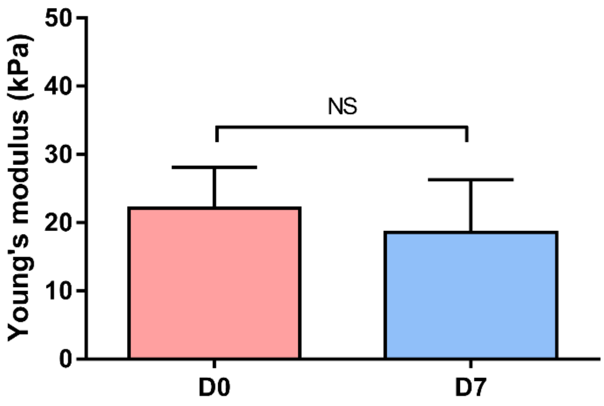

Figure S9. Average Young's modulus of the cell-laden hydrogels on Day 0 and Day 7 of culture. "NS" indicates no statistical significance ($P > 0.05$).

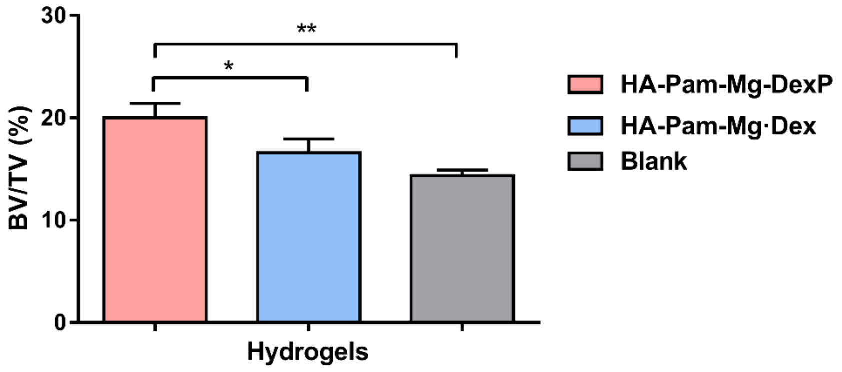

Figure S10. Quantitative bone volume analysis (BV: bone volume; TV: tissue volume) (n = 3); $*p < 0.05$, $**p < 0.01$.

Table S1. The sequence of the primers and probes used for real-time PCR is listed. The primer and probe sequences of the osteocalcin and Runx 2 are proprietary (Applied Biosystem) and not disclosed.

Gene	Forward primer	Reverse primer	Probe
**GAPDH**	AGGGCTGCTTTTA ACTCTGGTAAA	GAATTTGCCATGGG TGGAAT	CCTCAACTACATG GTTTAC
**COL I**	AGGACAAGAGGC ATGTCTGGTT	GGACATCAGGCGCA GGAA	TTCCAGTTCGAGT ATGGC
**ALP**	CGGAACTCCTGAC CCTTGAC	TGTTCAGCTCGTAC TGCATGTC	TCGAAGAGACCC AATAGGT
